# Effects of surgeon variability on oncologic and functional outcomes in a population-based setting

**DOI:** 10.1186/1471-2490-14-25

**Published:** 2014-03-06

**Authors:** Sigrid Carlsson, Anders Berglund, Daniel Sjoberg, Ali Khatami, Johan Stranne, Svante Bergdahl, Pär Lodding, Gunnar Aus, Andrew Vickers, Jonas Hugosson

**Affiliations:** 1 Urology Service at the Department of Surgery, Memorial Sloan-Kettering Cancer Center, 307 E. 63rd St, 2nd floor, New York, NY 10065, USA; 2 Department of Urology, Sahlgrenska Academy at the University of Göteborg, Göteborg, Sweden; 3 Department of Surgical Sciences, Uppsala University, Uppsala University Hospital, Uppsala, Sweden; 4 Department of Epidemiology and Biostatistics, Memorial Sloan-Kettering Cancer Center, New York, USA; 5 Department of Urology, Carlanderska hospital, Göteborg, Sweden

**Keywords:** Prostate cancer, Radical prostatectomy, Erectile function, Urinary function

## Abstract

**Background:**

Oncologic and functional outcomes after radical prostatectomy (RP) can vary between surgeons to a greater extent than is expected by chance. We sought to examine the effects of surgeon variation on functional and oncologic outcomes for patients undergoing RP for prostate cancer in a European center.

**Methods:**

The study comprised 1,280 men who underwent open retropubic RP performed by one of nine surgeons at an academic institution in Sweden between 2001 and 2008. Potency and continence outcomes were measured preoperatively and 18 months postoperatively by patient-administered questionnaires. Biochemical recurrence (BCR) was defined as a prostate-specific antigen (PSA) value > 0.2 ng/mL with at least one confirmatory rise. Multivariable random effect models were used to evaluate heterogeneity between surgeons, adjusting for case mix (age, PSA, pathological stage and grade), year of surgery, and surgical experience.

**Results:**

Of 679 men potent at baseline, 647 provided data at 18 months with 122 (19%) reporting potency. We found no evidence for heterogeneity of potency outcomes between surgeons (P = 1). The continence rate for patients at 18 months was 85%, with 836 of the 979 patients who provided data reporting continence. There was statistically significant heterogeneity between surgeons (P = 0.001). We did not find evidence of an association between surgeons’ adjusted probabilities of functional recovery and 5-year probability of freedom from BCR.

**Conclusions:**

Our data support previous studies regarding a large heterogeneity among surgeons in continence outcomes for patients undergoing RP. This indicates that some patients are receiving sub-optimal care. Quality assurance measures involving performance feedback, should be considered. When surgeons are aware of their outcomes, they can improve them to provide better care to patients.

## Background

Radical prostatectomy (RP) with curative intent is the most common treatment for men with localized prostate cancer [1]. A prospective, randomized Swedish trial demonstrated that surgery provides a survival benefit in men with mainly clinically diagnosed, palpable tumors when compared to watchful waiting [2]. However, surgical treatment is associated with long-term morbidity, mainly erectile dysfunction (ED) and urinary incontinence, which affect a patient’s quality of life (QoL) substantially [3,4].

Previous studies performed in high-volume referral centers in the United States (US) have shown that both oncologic [5] and functional outcomes [6,7] after RP vary between surgeons to a greater extent than is expected by chance. In the present validation study, we sought to explore whether such heterogeneity exists in long-term functional and oncologic outcomes among surgeons in a European center who operate in a population-based academic setting. Another rationale for the present study is that there is limited data on variability in patient-reported outcomes (PRO). Therefore, the present study provides a unique opportunity to explore any variability in outcomes as assessed by validated PRO instruments.

## Methods

The original database consisted of 1,447 men who were scheduled for open retropubic RP at Sahlgrenska University Hospital in Göteborg, Sweden, during the study period 2001–2008, when standardized data recording into a quality assurance database was performed.

The quality control program was approved by the Ethical Committee at Göteborg University in 2001. Patients were mailed questionnaires, a cover letter with information regarding the quality assurance program, and a statement of voluntary participation in the study.

Of the 58 patients excluded, 38 were enrolled in another clinical RP trial (LAPPRO) [8], 4 underwent surgery outside the university hospital, 7 did not undergo surgery (with 4 switching to radiotherapy and 3 receiving hormonal therapy), and 9 did not undergo surgery but may have had surgery elsewhere for unknown reasons (moved or loss-to-follow-up). This left 1,389 patients in the cohort. Surgery dates ranged from Jan 2, 2001 through July 16, 2008.

Biochemical recurrence (BCR) post-prostatectomy was defined as a prostate-specific antigen (PSA) value > 0.2 ng/mL with at least one confirmatory rise. Measurements were obtained from medical charts.

Of 1,389 patients, an additional 109 were excluded from analysis since they were never administered any questionnaires, leaving 1,280 patients available for analysis of functional outcomes. Patients responded to questionnaires regarding continence and potency on 4 occasions, approximately 2 weeks preoperatively and at 6, 18, and 36 months after surgery.

Patients were administered questionnaires assessing urinary continence using pads [9]. Continence was defined as no leakage or occasional leakage associated with physical activity requiring sporadic use of pads (score 0–1 on a 0–4 point scale, where 2–4 implies incontinence with regular use of pads).

Erectile function was assessed by the standardized International Index of Erection Function questionnaire IIEF-5 [10]. The IIEF-5 score consists of 5 items with 6 responses. The total score is the sum of the 5 items and ranges from 5–25, the higher the score the better potency. For patients missing responses for an item, the sum of the remaining items was used. Potency was defined as an IIEF-5 score of ≥ 17, which corresponds to Rosen et al.’s categorization of mild ED (17–21) and no ED (22–25) [10]. This also makes our study comparable to functional outcomes reported in an earlier study by Vickers et al. where postoperative potency was defined as 1 = normal, full erections and 2 = full, but diminished erections [6].

Missing data on potency status (potent/impotent) and continence (incontinent/continent) at 18 months were imputed following an algorithm assuming that few men regain and then lose function as well as that recovery of function can occur beyond 18 months. If a patient was missing a questionnaire at 18 months but reported potency at 6 months, he was assumed to be potent at 18 months. Comparably, a patient reporting impotence at 6 and 36 months was considered impotent at 18 months. A similar approach was used for the continence endpoint.

Patients reporting use of alprostadil injections for erectile aid were categorized as impotent (3 men preoperatively, 222 men at 18 months, and 198 men at 36 months). Men reporting use of PDE5-inhibitors were included in the analysis.

### Statistics

Data on a total of 25 individual surgeons working at the hospital during the study period were included in the database. We analyzed patient-reported functional outcomes for 9 surgeons who performed ≥ 20 surgeries during the study period. Data for these surgeons were entered both as random and fixed effects.

Logistic regression models were adjusted for age at surgery, PSA at diagnosis, pathologic stage (pT0, pT2, pT3), pathologic Gleason score (≤ 6, 7, ≥ 8), year of surgery, and surgical experience. We defined surgical experience as a variable that took into account both the surgeon’s prior experience, i.e., number of RPs before 2001, and the annual number of prostatectomies performed during the study period [6]. To statistically test for heterogeneity, a random effect following an inverse Gaussian distribution was included in the model.

The logistic regression model used to predict the probability of potency at 18 months was restricted to men who were potent preoperatively and also adjusted for the IIEF-5 score as a continuous variable. The model for continence at 18 months was restricted to men who were continent preoperatively. Since there have been changes in patient characteristics as well as operative technique, we also included year of surgery as a covariate. Nerve sparing status was not included in the model since it is a surgical decision. Take the case of two surgeons, one of whom only spared nerves if the cancer was very low risk and accordingly resected far from the neurovascular bundles, the other of whom only resected nerves for advanced disease. Overall, the former surgeon would have far lower potency rates, but adjusting for nerve sparing would lead to higher apparent rates.

A log-logistic distributed parametric regression survival model was used to model BCR rates following adjustment for the same covariates as for functional outcomes. To statistically test for heterogeneity, a shared frailty survival model was fitted.

For each surgeon studied, forest plots were created for the adjusted predicted probability of potency and continence, respectively, using the mean value for the covariates from the fixed regression model with a 95% confidence interval.

A scatter plot was created for adjusted rates of continence and potency with the size of each surgeon’s data point proportional to the number of RPs (prior experience and during the study period) performed by each surgeon. Spearman’s rank correlation was applied to test the correlation between surgeons’ adjusted probabilities of potency and continence at 18 months as well as BCR.

Statistical analyses were performed using Stata v. 12.0 (Stata Corp, College Station, TX, USA).

## Results

Table 1 shows clinical characteristics for all 1,280 men who underwent RP. The median age at surgery was 64 years. Crude functional outcome rates per surgeon along with prior experience and the number of cases operated upon during the study period are shown in Table 2. Nine surgeons performed ≥ 20 surgeries during the study period, with a maximum of 248 cases. The prior experience of these surgeons ranged between 0 and 360 cases (Table 2).

**Table 1:** Clinical characteristics for all patients who underwent radical prostatectomy

		**All patients N = 1,280**	
**Age at surgery, median years (IQR)**		64	(60,67)
**PSA at diagnosis, median (IQR) ng/mL** (*Missing n=3*)		6.3	(4.5,9.8)
**Follow-up for biochemical recurrence, median years (IQR)**		5.28	(3.59-6.83)
		**n**	**(%)**
**Pathological Gleason score** (*Missing n=2*)	≤ 6	593	(46.5)
	7	624	(48.9)
	≥ 8	59	(4.6)
	No tumor found	2	-
**Nerve-sparing surgery** (*Missing n=3*)	No	625	(48.9)
	Unilateral	196	(15.3)
	Bilateral	456	(35.7)
**Surgical margins** (*Missing n=5*)	Positive	333	(26.1)
	Negative	942	(73.9)
**Seminal vesicle invasion** (*Missing n=16*)	Yes	109	(8.6)
	No	1155	(91.4)
**Pathological stage** (*Missing n=7*)	pT0	2	(0.2)
	pT2	906	(71.2)
	pT3	365	(28.7)
**Preoperative erectile function** (*Missing n=241*)	Potent	679	(65.4)
	Impotent	360	(34.7)
**Postoperative erectile function at 18 months** (*Missing n=633*)	Potent	122	(18.9)
	Impotent	525	(81.1)
**Preoperative urinary function** (*Missing n=202*)	Continent	1053	(97.7)
	Incontinent	25	(2.3)
**Postoperative urinary function at 18 months** (*Missing n=301*)	Continent	836	(85.4)
	Incontinent	143	(14.6)

PSA = prostate-specific antigen, IQR = Inter Quartile Range.

**Table 2:** Unadjusted functional outcome rates per surgeon (among preoperatively potent and continent men) in relation to prior experience and number of cases performed during study period

**Surgeon number**	**Prior experience, number of RPs performed before study (n)**	**Number of cases during study period (n)**	**Potent at 18 months, n (%)**	**Impotent at 18 months, n (%)**	**Missing potency information at 18 months, n (%)**	**Continent at 18 months, n (%)**	**Incontinent at 18 months, n (%)**	**Missing continence information at 18 months, n (%)**
**1**	32	94	4 (9.1)	38 (86.4)	2 (4.5)	56 (78.9)	14 (19.7)	1 (1.4)
**2**	120	248	20 (15.9)	98 (77.8)	8 (6.3)	171 (86.8)	15 (7.6)	11 (5.6)
**3**	2	104	12 (20.0)	45 (75.0)	3 (5.0)	69 (76.7)	14 (15.6)	7 (7.8)
**4**	360	156	20 (24.1)	59 (71.1)	4 (4.8)	115 (84.6)	14 (10.3)	7 (5.1)
**5**	13	239	25 (19.4)	98 (76.0)	6 (4.7)	157 (83.1)	18 (9.5)	14 (7.4)
**6**	115	138	16 (19.3)	64 (77.1)	3 (3.6)	80 (66.1)	27 (22.3)	14 (11.6)
**7**	67	97	9 (16.1)	46 (82.1)	1 (1.8)	69 (82.1)	11 (13.1)	4 (4.8)
**8**	2	75	7 (20.6)	25 (73.5)	2 (5.9)	43 (71.7)	12 (20.0)	5 (8.3)
**9**	0	49	4 (13.8)	22 (75.9)	3 (10.3)	21 (58.3)	10 (27.8)	5 (13.9)

RPs = radical prostatectomies.

Regarding functional outcomes, a total of 1,039 men responded to the IIEF-5 questionnaire, with 679 reporting being potent preoperatively (65%). Data were available for 647 (95%) patients at 18 months with 122 (19%) reporting potency (without alprostadil).

Crude potency rates at 18 months were higher for men who underwent bilateral nerve-sparing RP (31.8%) and for those < 60 years of age (45.7%) (data not shown).

Of the 1,078 patients who responded preoperatively to the continence question, 25 were considered incontinent (2%). Of the 979 (91%) patients responding at 18 months, 836 were continent (85%). The degrees of incontinence at 18 months among preoperatively continent men were as follows: never urinary leakage (39.0%), sometimes urinary leakage when coughing, sneezing or performing physical exercise with sporadic use of pads (46.4%), regular use of pads but they are not always wet (9.4%), regular use of pads that are wet (3.3%), and constant urinary leakage (1.7%).

**Figure 1:**
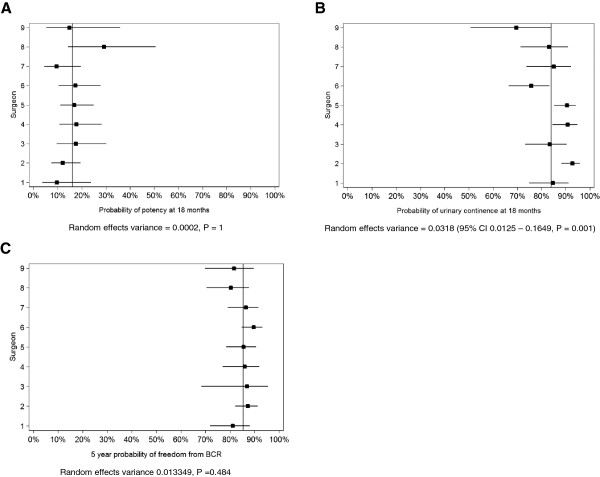
**Forest plots for the probability of functional and oncologic outcomes by surgeon. A**. Probability of potency at 18 months postoperatively. **B**. Probability of continence at 18 months postoperatively. **C**. 5-year probability of freedom from biochemical-free recurrence. Proportions (squared dots) and 95% confidence intervals (horizontal lines) are adjusted for patients with the mean level of the covariates: age at surgery, PSA at diagnosis, pathological stage, pathologic Gleason score, year of surgery and surgical experience. The vertical line represents the mean adjusted probability for all surgeons. Potency was defined as an IIEF-5 total score ≥ 17, with alprostadil-users defined as impotent. Continence was defined as urinary control with no leakage or sporadic use of pads due to leakage associated with physical activity.

Between-surgeon variation in functional outcomes is shown in the forest plots for potency in Figure 1A and continence in Figure 1B. For potency at 18 months, we found no evidence for heterogeneity between surgeons (random effects variance = 0.0002, P = 1). For continence at 18 months, there was a statistically significant heterogeneity between surgeons, (random effects variance = 0.0318; 95% CI 0.0125 – 0.1649, P = 0.001) with continence rates varying from 70 to 93% between surgeons. For BCR at a mean follow-up of 5.12 years (SD 0.07), heterogeneity between surgeons was not observed (random effects variance = 0.0133, P = 0.484) (Figure 1C).

**Figure 2:**
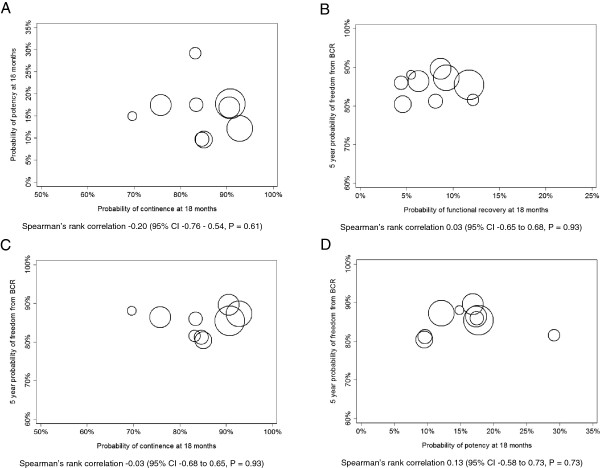
**Scatter plots. A**. Adjusted probability of continence and potency at 18 months postoperatively. **B**. Adjusted probability of functional recovery and 5-year probability of freedom from biochemical recurrence at 18 months postoperatively. **C**. Adjusted probability of continence and 5-year probability of freedom from biochemical recurrence at 18 months postoperatively. **D**. Adjusted probability of potency and 5-year probability of freedom from biochemical recurrence at 18 months postoperatively. Each circle represents a single surgeon and the size is proportionate to the surgeon’s prior experience and the number of radical prostatectomies that surgeon performed during the study period.

The correlation between surgeons’ adjusted probabilities of potency and continence was not statistically significant (Spearman’s rho -0.20, P = 0.61) (Figure 2A).

Postoperatively, the 5-year probability of freedom from BCR adjusted for case mix was 86%. We observed a weak and non-significant association between surgeons’ adjusted probabilities of functional recovery (i.e., both potent and continent) and 5-year probability of freedom from BCR (P = 0.9) (Figure 2B). The present findings were not altered when the correlation between functional recovery and BCR was assessed separately; continence and potency, respectively (Figure 2C and 2D).

## Discussion

In the present validation study, we explored whether heterogeneity in long-term functional outcomes in a European academic center is comparable to that previously reported in a very high-volume referral center in the US [6]. Our data confirms prior studies on the existence of surgeon heterogeneity for continence, but not for potency [5-7].

Continence rates were similar when comparing our present study to the Vickers et al. study in which 1,910 patients were treated with RP by 1 of 11 surgeons between 1997 and 2007 at a high-volume US referral center [6], with a rate of 85% at 18 months vs. 83% at 12 months, respectively. However, potency rates were lower in the present study, with a rate of 19% at 18 months compared to 43% at 12 months in Vickers et al. Overall, the rate of impotence at 18 months postoperatively was high in the present study, with less than two-thirds of patients being potent preoperatively. The lower rate of potency is most plausibly explained by the fact that the men in this study were considerably older, with a median age of 64 years, as compared to the median age of 58 years in the Vickers et al. study. Another possible explanation is that the study cohort was derived from a population-based sample in an area where active surveillance is frequently used, implying that only the most aggressive tumors are treated; this likely results in more advanced tumor features leading to more radical surgery with wider resection and lower potency rates. This is exemplified in that only one third of patients underwent a bilateral nerve-sparing procedure. Additionally, center-specific differences in indication for nerve-sparing procedures may explain these results.

A major strength of the current study is that potency and continence outcomes were assessed from patient-administered questionnaires in contrast to the study by Vickers et al., where the same outcomes were evaluated by the treating surgeon. PROs provide an assessment of functional outcomes from the patient’s perspective. Therefore, collecting data from the patient may protect against bias due to patients minimizing symptoms when they are asked by the treating physician directly. Published rates of impotence after RP based on physician reports may be underestimates, since patients may not report this side effect accurately and completely to their doctor [11].

Furthermore, the patient-reported potency rate in the present study is in line with other population-based series on unselected patients in the literature [11-15]. In the Scandinavian Prostate Cancer Group Study 4 (SPCG-4) trial, the prevalence of ED 12 years after RP was 84% [3].

We found no evidence of heterogeneity in potency by surgeon. Our failure to replicate a prior finding of heterogeneity for potency outcomes [6] may be related to a floor effect of low overall rates as well as limited power (9 surgeons). Other possible explanations are that potency postoperatively is more a question of inherent patient factors or also dependent upon the individual decision of the surgeon and patient to elect for a nerve-sparing procedure.

For continence outcomes at 18 months, we found evidence of significant heterogeneity between surgeons. The difference was quite remarkable (7%–30% incontinence rates). This finding is in line with a previous US study. Using the SEER-Medicare database (patients ≥ 65 years old); Begg et al. found that case mix-adjusted incontinence rates 1 year after RP were lower in very high-volume hospitals than in low-volume hospitals. They also studied, in detail, long-term incontinence in patients for 159 surgeons in the 2 highest-volume categories (20–32 RPs per surgeon and 33–121 RPs per surgeon during the study period) and noted significant surgeon-to-surgeon variations in outcome [7].

Urinary incontinence and impotence after RP have a major impact on QoL [4] and these side effects, especially in the long-term, are one of the major drawbacks of PSA screening [16]. In the present study the average incontinence rate was 15%, which is very similar to the 17% reported by Vickers et al. [6]. The large variation between surgeons suggests a need for formal quality assurance programs and performance feedback systems. Surgeons need to know their results so that they can evaluate and find ways to improve them [17].

Previous studies have shown heterogeneity between surgeons regarding not only functional outcomes but also risk for BCR and clinical recurrence [5,6,18]. In particular, Vickers et al. found that functional preservation did not come at the expense of cancer control [6]. Indeed, there was a positive correlation between oncologic and functional outcomes, suggesting that both are markers of surgical quality. We were not able to replicate this finding, possibly due to a floor effect for potency.

## Conclusion

RP is associated with long-term side effects such as incontinence and ED. In this study we replicated prior findings that individual surgeon technique is related to the risk of permanent incontinence; we were unable to replicate findings relating surgical technique to erectile dysfunction and oncologic outcome. Quality assurance measures involving performance feedback should be considered. When surgeons are aware of their outcomes, they can improve them to provide better care to patients.

## Abbreviations

BCR: Biochemical recurrence; ED: Erectile dysfunction; PSA: Prostate-specific antigen; RP: Radical prostatectomy; PRO: Patient-reported outcomes; QoL: Quality of life; IIEF-5: International index of erectile function 5 questionnaire; SPCG-4: Scandinavian prostate cancer group study 4.

## Competing interest

None of the authors have any competing interest to declare.

## Authors’ contributions

AV, JH and SC conceived the study concept and design. The data acquisition was performed by JH and SC. AV supervised the statistical methods and analyses. AB and DS carried out the statistical analyses and plotted the figures. SC and AV drafted the manuscript. SC, JH and AV obtained funding. AK, JH, SB, PL, GA and JH performed the radical prostatectomies in the study. All authors interpreted the data and critically revised the manuscript for important intellectual content. All authors read and approved the final manuscript.

## Pre-publication history

The pre-publication history for this paper can be accessed here:

http://www.biomedcentral.com/1471-2490/14/25/prepub
